# The Atrioventricular Coupling in Heart Failure: Pathophysiological and Therapeutic Aspects

**DOI:** 10.31083/j.rcm2505169

**Published:** 2024-05-14

**Authors:** Vito Di Terlizzi, Roberta Barone, Nicola Di Nunno, Gianmarco Alcidi, Natale Daniele Brunetti, Massimo Iacoviello

**Affiliations:** ^1^Cardiology Unit, University Policlinic Hospital Riuniti, 71122 Foggia, Italy; ^2^Department of Surgical and Medical Sciences, University of Foggia, 71122 Foggia, Italy

**Keywords:** atrio-ventricular coupling, heart failure, atrial failure, strain imaging

## Abstract

For a long time, the study of heart failure focused on single heart chamber disease. There is, instead, growing attention on the interplay between the atria and the ventricles during the cardiac cycle and on the consequences of an altered chamber coupling on global heart performance and heart failure. This review aimed to explore the principles of atrioventricular (AV) function and coupling of the left heart and the consequences that their disruption could have in several diseases. Furthermore, we will examine echocardiographic tips for analyzing the chamber function and the AV coupling. Finally, we will explore the most recent pharmacological acquisitions and the device therapies we have for use.

## 1. Introduction

The atria have a crucial role in left ventricle (LV) filling and global heart performance, and they have a dynamic interaction with ventricular diastole and systole. The atrial performance results from atrial compliance, ventricular relaxation, and transmitral pressure gradient [1, 2]. It can be assessed by measuring the reservoir, which expresses the combination of conduit and contraction phases and may detect subclinical left atria (LA) myocardial dysfunction even before structural changes occur [3]. Conditions impairing any phase of atrial function may affect global cardiac performance, leading to symptoms and worsening outcomes, a pathology currently known as atrial failure [2]. The atrial strain evaluation. In this setting, atrioventricular (AV) coupling is also essential for synchronizing atrial cycle phases to LV diastole [4]. It can play a very relevant role in patients affected by heart failure (HF).

This review aimed to focus on the main pathophysiological aspects of atrial dysfunction and AV uncoupling as well as the diagnostic and therapeutic implications.

## 2. Atrio-ventricular Function

### 2.1 Atrial Function 

The atria are two chambers located posteriorly and above the ventricles. They are divided by the interatrial septum and receive the blood from the pulmonary veins (the left atrium) and the cava veins (the right atrium). The left atrial appendage, a trabeculate independently attached structure with high anatomical variability, has an important endocrine function. The atria have a crucial role in LV filling and global heart performance, and they interact dynamically with ventricular diastole and systole [5].

Recently, atrial cardiomyopathy has been distinguished from atrial failure, the last referring to the functional consequences of any atrial condition, including but not restricted to primary atrial diseases [2]. According to the definition of Bisbal *et al*. [2], it is defined as “any atrial dysfunction (anatomical, mechanical, electrical, and/or rheological, including blood homeostasis) causing impaired heart performance and symptoms, and worsening quality of life or life expectancy, in the absence of significant valvular or ventricular abnormalities” [6]. Atrial dysfunction could be related to different etiologies such as electrical interatrial and AV dyssynchrony, booster-pump failure (determined by atrial fibrosis, ischemia and disorganized atrial activation), reservoir dysfunction and conduit dysfunction (caused by atrial dilatation, spherical deformation and altered pressure gradient). Moreover, these conditions could lead to atrial fibrillation, which, in turn, could further increase the probability of suboptimal LV filling, increased filling pressure and pulmonary pressure. In this perspective, atrial failure may cause HF symptoms standalone, in a condition of heart failure with preserved ejection fraction (HFpEF), or aggravate or decompensate HF with reduced LV ejection fraction (HFrEF).

### 2.2 Ventricular Function 

The two ventricles are the power engine of the heart, responsible for creating the pressure needed for the cardiac work and generating the cardiac output. The mechanical action of the LV occurs by a dual motion. First, the constriction of the circular muscle layers reduces the diameter of the chamber, progressing from apex to base. Then, the contraction of the spiral muscles pulls the mitral valve ring toward the apex, thereby shortening the long axis. The conical shape of the lumen gives the LV a smaller surface-to-volume ratio than the right ventricle (RV) and contributes to the ability of the LV to generate high pressures [5].

On the contrary, the mechanical action of the RV resembles that of a bellows used to fan a fire. The mechanism of emptying the RV involves three motions. First, the longitudinal axis of the RV shortens when spiral muscles pull the tricuspid valve ring toward the apex. Second, the free wall of the RV moves toward the septum in a bellows-like motion. Third, the contraction of the deep, circular fibres of the LV forces the septum into a convex shape so that the septum bulges into the RV. This bulging of the septum stretches the free wall of the RV over the septum. These three motions are well suited for the ejection of a large volume but not for developing a high pressure [7].

In the diastolic phase, ventricular muscle relaxation leads to repositioning the AV plane to the initial position. The preserved elastic and functional properties of the ventricular as well as the atrial muscle grant the two chambers to return to their initial shape. In this phase, repositioning the AV plane and re-shaping the atrium and the ventricle allows the re-opening of the AV valves and the rapid initial ventricle filling. The AV valve opening starts the conduit phase of the atrial cycle, during which a minimum amount of blood flows from the central vein to the atria and then through the valves to the ventricles [6].

### 2.3 Atrio-ventricular Junction 

The atrial and ventricular chambers are joined through the AV junction, which is made in the prevalence of fibrous tissue, involving the mitral annulus, the tricuspid annulus, the fibrous trigone and the semilunar valves. The fibrous composition confers more resistance to traction and allows electrical isolation. The mitral and tricuspid valve leaflets originate from the circumferences of the AV valves. The atrial and myocardial fibres are inserted at the level of the AV junction into the mitral and tricuspid circumferences [8]. The excursion of the AV junction is crucial for cardiac pump function. During the cardiac cycle, the AV junction moves towards the apex and returns to the original position in proto-diastole. The mitral and tricuspid apparatus, i.e., annulus, leaflets, chordae tendineae, and papillary muscles, actively contribute to the AV junction motion during the cardiac cycle [9].

### 2.3 Constant-volume Heart Model 

The physiological model of cardiac function is the constant-volume attribute of the four-chambered heart, which states that the total volume of the four-chambered heart (i.e., the contents of the pericardial sack) does not vary throughout the cardiac cycle [10]. The constant-volume attribute has immediate and direct consequences regarding cardiac function, particularly during diastole. The critical point of the constant-volume model is that as the ventricles empty, the atria fill up, resulting in a simultaneous reciprocation of volumes.

During ventricular systole, the orientation of the myocardial fibres guarantees a longitudinal shortening, which leads to the movement of the plane of the valve towards the heart apex, in addition to a radial movement of the fibres. The relevance of preserving an AV plane function (and so the AV coupling) can be understood through the mechanism of the heart’s reciprocal filling and emptying. The movement of the AV plane is essential not only for the ejection of blood but also for the filling of the atria [11]. During ventricular systole, the movement of the plane towards the apex causes a drop in atrial pressures, and the blood is aspirated into the atria from the cava and pulmonary veins. Thus, when the ventricle empties, the atrium fills up. Consequently, the heart maintains a constant volume and may save energy for the heart.

**Fig. 1.**
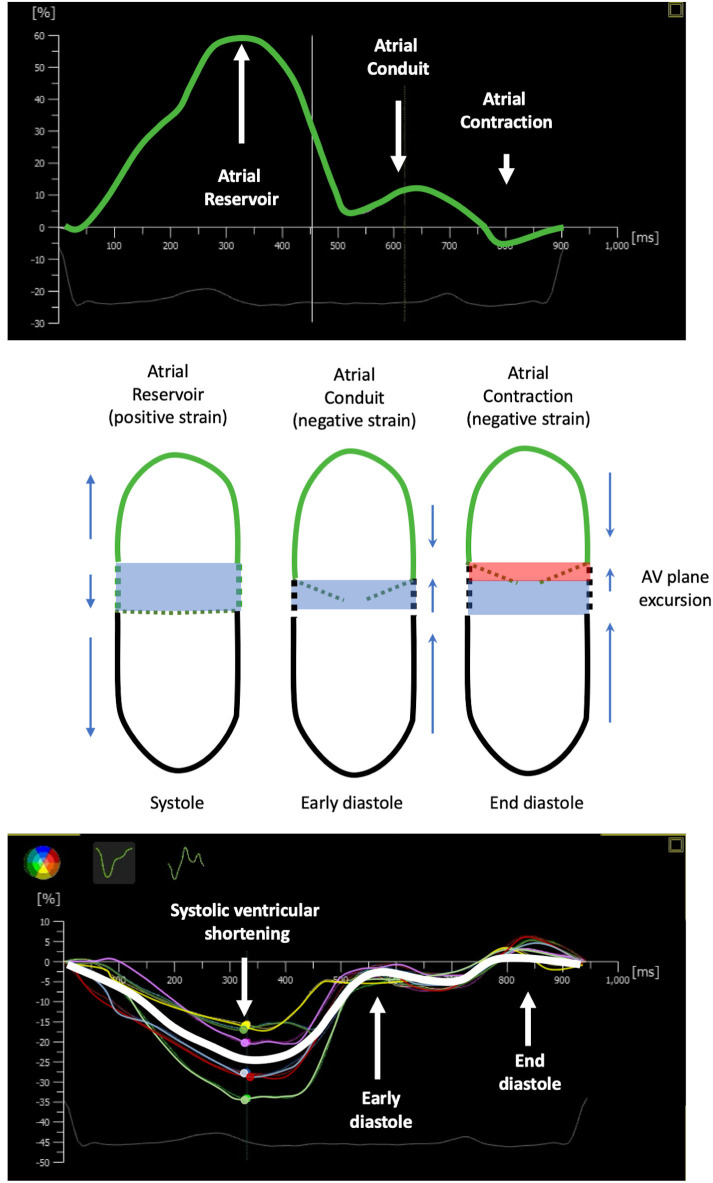
**Atrial and ventricular strain and atrio-ventricular coupling**. Left atrial and ventricular strain of systolic and diastolic function. The figure provides a comprehensive overview of atrial and ventricular strain during systolic and diastolic functions. The upper portion illustrates the left atrial strain in normal subjects, segmented into three distinct phases: Reservoir, Conduit, and Contraction. Meanwhile, the lower segment of the figure depicts the left ventricular longitudinal strain in normal subjects, categorized into systole, early diastole, and end diastole. A striking symmetry and close interrelation between atrial and ventricular strain are evident throughout the illustration. At the heart of the figure, the reciprocal interaction between atrial and ventricular dynamics during the cardiac cycle is showcased, influencing the AV plane excursion. This intricate interplay results in the transfer of volumes between the two chambers, a process central to understanding the complex mechanics of the cardiovascular system. 2DS, two-dimensional speckle tracking; AV, atrioventricular.

This theory has yet to be proven. Carlsson *et al*. [9], in their study, analyzed the contribution of the AV plane displacement through cardiac magnetic resonance, and their analysis showed that the AV plane movement contributes 60% to the entire stroke volume, especially in young people, where the longitudinal movement is favored over the radial one. According to this heart model, the AV junction movement during the cardiac cycle is crucial for heart function and allows the prediction of various aspects of cardiac pump function (Fig. 1).

## 3. Atrio-ventricular Coupling

Timely AV coupling is essential for synchronising atrial cycle phases to LV diastole. LA inflow from the pulmonary veins occurs during LV systole and isovolumetric relaxation (reservoir function), and accounts for approximately 40% to 50% of the LV stroke volume [12]. Passive blood transfer during LV diastole (conduit function) constitutes approximately 20% to 30% of stroke volume. It precedes the active atrial contraction (booster-pump function), which transfers the remaining volume (20%–30%) to the LV. The contraction of the atria normally makes only a minor contribution to the filling of the two ventricles when the subject is at rest. In contrast, it is a useful safety factor during tachycardia, when the diastolic interval—and thus the time for passive filling—is short, and when diastolic ventricular function is impaired.

The performance of the reservoir phase is determined by atrial compliance, ventricular relaxation, and transmitral pressure gradient. Conditions impairing any atrial function, especially mechanical alterations leading to an abnormal pressure-volume relationship, may affect global cardiac performance, leading to symptoms and a worse outcome.

The interplay between LA and LV functions throughout the cardiac cycle (AV coupling) is crucial in several pathophysiological conditions [13]. The LA and LV functions are strictly connected each other. As previously demonstrated, providing that the atria and the ventricles are linked through the AV plane, a reduction in ventricular contraction corresponds to a worsening of atrial function at higher heart rate [14]. A first hypothesis is that the heart rate dependent worsening of both left chambers could be related to atrial dysfunction. Indeed, a decrease in the atrial contribution to LV filling (just as the reduction of AV plane movement) could determine a reduced longitudinal ventricular shortening. Another hypothesis is that the heart rate dependent reduction of LV function could lead to increased LV filling pressures and elevated muscular stiffness which are responsible for the occurrence of atrial dysfunction. These changes can lead to a reduced excursion capacity of the AV plane and a decreased capacity of modulating its filling during cardiac cycle. Otani *et al*. [15] suggested that a compensatory increase may be observed in LA booster pump function in patients with mild and moderate diastolic dysfunction although a significant reduction in those with severe diastolic dysfunction. LA myocardial fibrosis could even play a role in this context, precluding the compensatory increase in the active LA contraction. Indeed, in early diastolic dysfunction, decreased ventricular compliance and elevated filling pressures decrease early transmitral passive diastolic flow, thus atrial pump function increases to compensate for LV filling. As LV distensibility further decreases, atrial pressure increases to maintain cardiac output, until LA compliance also decreases. The reduction in ventricular contraction as well the decreased atrial function lead to what we could refer to as “AV plane dysfunction” or AV uncoupling. In this sense, the dysfunction of the two chambers is strictly related each other and can reciprocally influence both functions determining a condition of AV function worsening and increased filling pressures.

## 4. Atrio-ventricular Uncoupling: from Mechanical to Electrical Uncoupling

According to the model of cardiac pump function and the AV junction excursion during the cardiac cycle, it is possible to imagine two different mechanisms that can lead to AV uncoupling. In the first case, the AV junction excursion during systole could be reduced or even abolished, altering the principal mechanism responsible for ventricular ejection and filling. In the second one, the PR interval prolongation could be responsible for the dyssynchrony between the atrial and the ventricular systole.

### 4.1 Mechanical AV Uncoupling in Heart Failure

As focused on before, the AV junction excursion is the leading heart movement responsible for cardiac pump function. The extent of displacement firstly reduces with older age. Some studies have demonstrated that the measures of AV junction excursion (as estimates of ventricular function) tend to reduce in advanced age, i.e., mitral annular plane systolic excursion (MAPSE) [16]. It is also demonstrated for longitudinal strain measures, at least for LV [17]. Considering that ejection fraction (EF) is often preserved, we can assume that with ageing, there is a progressive reduction of the ventricular longitudinal contraction balanced by circumferential and radial deformation [18]. As the systolic excursion of the AV junction is reduced, the diastolic relaxation is decreased, too. In the elderly, the E wave measured with Pulsed Wave (PW) Doppler and the e’ wave measured with Tissue Doppler Imaging (TDI) is reduced, so the AV uncoupling leads to the impairment of LV filling in older age [19]. The natural ageing of the myocardial muscle seems to be associated with this trend. Moreover, in several pathologies, the AV junction excursion could be further or less affected.

HF is characterized by the inability of the heart to create an adequate cardiac output or the need to increase filling pressures to do it. All systolic and diastolic function indices are reduced in HF with reduced EF. Together with reduced EF, MAPSE, TDI measures and LV global longitudinal strain (GLS) are reduced too [20]. The measures of atrial function are even decreased, generally associated with an altered diastolic function obtained by atrial longitudinal strain. In this case, however, considering that the heart function is globally affected, the AV uncoupling may not be the primary mechanism of the disease.

Nevertheless, it is interesting to note what happens at higher frequency rates. As demonstrated in some studies, patients affected by HF poor tolerate higher frequencies. Indeed, at higher stimulation rates, ventricular and atrial strain measures tend to reduce together with a worsening of the E/e’ ratio and diastolic function indices [21].

On the contrary, patients with HFpEF have an EF ≥50%, according to recently published European Society of Cardiology (ESC) guidelines. In this case, LV systolic function is preserved, while the LV diastolic function is altered. However, this could not be true at all. In fact, patients affected by HFpEF present reduced systolic AV junction excursion, even with a preserved global EF, as demonstrated by decreased values of M-mode parameters as well as TDI values and longitudinal strain measures. Instead of an altered AV coupling, the EF could probably be preserved thanks to higher radial and circumferential contraction, that allow to preserve the global volumes reduction during systole. This idea is also supported by the fact that fibres responsible for longitudinal and radial contraction are different, as demonstrated by heart dissections [22].

Moreover, some measures, i.e., the longitudinal peak systolic velocity, correlate with brain-type natriuretic peptide (BNP) levels better than EF [23]. Considering TDI measures, longitudinal peak velocities are reduced in aortic stenosis, aortic regurgitation and mitral regurgitation despite the preserved EF [24, 25, 26]. Nagueh *et al*. [27] have also demonstrated that reduced peak TDI velocity values could be found earlier in patients affected by hypertrophic cardiomyopathy and could be used for early identification and differential diagnosis. In hypertension and diabetes, TDI at the bases is useful for early detection of impaired longitudinal function, and Fang *et al*. [28] have demonstrated that reduced longitudinal contraction is compensated by increased radial contractility [29].

As demonstrated in several studies, LV GLS is also altered in patients with HFpEF. A sub-analysis of the RELAX trial (Effect of phosphodiesterase-5 inhibition on exercise capacity and clinical status in heart failure with preserved ejection fraction: a randomized clinical trial) also demonstrated that LV GLS correlates well with BNP values and presents a trend towards small left atrial volumes and E/A ratios. In the TOPCAT study (Spironolactone for Heart Failure with Preserved Ejection Fraction), this parameter was also associated with the outcome of cardiovascular (CV) death, HF hospitalisation and aborted cardiac arrest [30].

As demonstrated in several studies, left atrial strain is another valuable measure for evaluating patients affected by HFpEF. Reddy* et al*. [31] have demonstrated that left atrial strain strongly correlates with HF (if compared with normal subjects), even better than other measures such as left atrial volume index (LAVI), E/e’ or LV hypertrophy [31], and with atrial size and volume too.

LV GLS is also used for early evaluation of cancer therapy–related cardiac dysfunction. It is well demonstrated that the systematic evaluation of LV GLS in patients with cancer treated with potentially harmful chemotherapies helps recognise LV systolic dysfunction early [32].

All these data are well consistent with the idea that AV junction excursion, as the principal heart motion responsible for cardiac pump function in healthy people, is altered in several pathologies, and that is one of the first LV systolic components to have deteriorated. Atrial function and ventricle function are strictly related to each other through the AV junction. So, its altered movement is an expression of impaired ventricular and atrial function and ultimately of their relationship. From this perspective, we can affirm that AV uncoupling is not only a temporal and electrical pathology but can also be interpreted as a mechanical dysfunction, which could be part of a broad spectrum of heart pathologies.

### 4.2 Electro-mechanical AV Uncoupling: PR Prolongation

In the case of PR prolongation, the atrial systole occurs much earlier than ventricular systole, reducing the efficacy of the atrial contribution to the stroke volume. Indeed, premature atrial contraction is associated with the fusion of the E and the A waves [33, 34], which leads to a shorter LV filling time. Moreover, the atrial systole ends during the ventricular diastole instead of at the beginning of the ventricular systole, resulting in a desynchronization with the ventricular contraction. In this case, the absence of the ventricular myocardial contraction makes the mitral apparatus unable to close, leading to late-diastolic mitral regurgitation powered by the transient V-A positive pressure gradient [35, 36]. This is responsible for further reduced LV filling, decreased LV preload and reduced stroke volume (according to the Frank-Starlin mechanism). A marked PR prolongation (>300 msec) can also cause the atrial contraction to fall during the ventricular systole when AV valves are closed. It can cause several increases in atrial pressures, followed by increased pulmonary capillary wedge pressures. This situation may result in a pseudo-pacemaker syndrome characterized by dyspnea and retrograde blood flow in jugular veins.

PR prolongation is generally not associated with increased CV risk in common healthy people. However, in individuals who already have comorbidities and/or CV disease, PR prolongation worsens the prognosis. First, it raises the risk for atrial fibrillation in patients with stable artery coronary disease and/or hypertension (hazard ratio (HR) 1.2–1.3 in Health ABC (The Health, Aging, and Body Composition (Health ABC) Study-Ground-Breaking Science for 25 Years and Counting) and Atherosclerosis Risk in Communities (ARIC) Study) [37, 38]. Moreover, several studies have demonstrated that AV desynchronization increases the risk of HF and LV dysfunction together with an increased risk of HF hospitalization (between 39 and 51%) [39, 40]. Long PR interval was also a marker of a more severe degree of AV block in some studies [41, 42]. Finally, in several studies, PR prolongation was associated with a 10% increase in all-cause mortality, and patients had a higher risk of mortality or HF in the sub-analysis of two cardiac resynchronization therapy (CRT) trials [43, 44].

### 4.3 Electro-mechanical AV Uncoupling: Atrial Fibrillation

Atrial fibrillation is a very common arrhythmia in adult patients. Many of them complain of symptoms of dyspnea and fatigue. Some patients present with normal EF and others with reduced EF secondary to tachycardia-mediated cardiomyopathy. In atrial fibrillation, the electrical disorganization of atrial rhythm totally compromises atrial systolic and diastolic function. Moreover, the irregular rhythm and the absence of atrial contraction may result in a reduced LV filling. These alterations end up in AV uncoupling, considering that the AV junction excursion is hardly decreased (having loosed the atrial contraction contribution) and it is mainly driven by ventricular systolic contraction. This aspect is confirmed by the reduction of some indices of ventricular longitudinal shortening and, ultimately, of AV junction excursion, i.e., the LV GLS. Agner *et al*. [45] have demonstrated, in fact, that LV GLS is reduced in patients with atrial fibrillation compared to control subjects. Moreover, reduced LA and LV GLS are independent predictors of atrial fibrillation, together with LA volume size [46]. It is also known that these aspects correlate with atrial fibrosis burden [47]. Atrial fibrosis, often associated with atrial fibrillation, could be responsible of a significant stiffness of the atrial chambers, thus affecting the cardiac cycle contraction and the AV junction excursion.

## 5. Echocardiographic Analysis

The AV junction excursion (and so the AV coupling) during the cardiac cycle can be derived through semi-quantitative echocardiographic parameters such as the analysis of the trans-mitral and trans-tricuspid blood flow spectrogram, the movement of the mitral and tricuspid valve plane during systole and the assessment of the LV and RV longitudinal strain.

### 5.1 Mitral Pulsed and Tissue Doppler Imaging

The trans-mitral blood flow spectrogram can be obtained by positioning the Pulsed Wave Doppler (PWD) box above the ventricular side of the mitral valve in a 4-chamb view. This enables the assessment of the AV mechanical coupling by evaluating the diastolic filling of the LV in terms of ventricular relaxation and atrial contribution to diastole. A normal flow through the mitral valve is characterized by a first positive peak, called E wave, which represents rapid diastolic filling and is determined by the pressure difference between LA and LV at the time of valve opening, a deceleration time (dT) that is the duration of rapid diastolic filling and a second peak, called A, which corresponds to the atrial contribution to diastole represented by its contraction. In normal conditions, the E/A ratio is > one. Any pathology affecting AV junction excursion can alter the PWD of mitral flow. The extent of this impairment is variable, and it can be associated with different degrees of diastolic dysfunction, which are frequently (but not invariably) associated with typical PWD patterns. In case of initial diastolic dysfunction (1st degree or “impaired relaxation”), there is a prolongation of relaxation times with a consequent dT increasing, E wave speed reduction and E/A ratio inversion. This pattern is commonly found in older patients and may indicate some form of LV filling and AV junction movement impairment, not necessarily diagnosing diastolic dysfunction. Various cardiovascular diseases, including arterial hypertension, hypertrophic cardiomyopathy, and myocardial infarction, may exhibit this pattern in mitral pulsed Doppler. With the diastolic disease progression, LA pressure rises, leading to higher E wave velocities and a pseudo-normalization of the E/A ratio (2nd degree diastolic dysfunction). Severe diastolic dysfunction, characteristic of advanced LV myocardium disease, is marked by a very high E wave velocity, and E/A ratio >2 (restrictive pattern or 3rd degree diastolic dysfunction). It is essential to note that these patterns are not necessarily associated with a degree of diastolic dysfunction, as described earlier. For instance, E/A wave inversion is considered para-physiologic in older patients, and in some cases, 1° grade diastolic dysfunction may present with an E/A ratio >1. Thus, diastolic dysfunction diagnosis and estimation necessitate a multiparameter approach, as outlined in ASE guidelines [27]. Nevertheless, it is worth mentioning that evaluating these parameters provides valuable insights into the interplay between the atrium and ventricle, the extent of AV junction movement impairment, and notably, how the heart attempts to maintain a constant intracardiac volume in the presence of impaired diastole, often seen in patients with heart failure.

The timing of E and A peak waves is also relevant. This interval is the diastasis; its duration varies depending on the heart rate. However, among the common frequencies, the fusion of the E and A peak waves or their desynchronization could stand for different degrees of AV block and, therefore, AV electrical uncoupling.

TDI analysis allows us to measure the mitral excursion velocity during the cardiac cycle. The rationale is that, in healthy hearts, the greater part of the left ventricular ejection and left atrial filling derives precisely from the downward movement of the mitral annulus towards the apex. The typical mitral TDI spectrum displays three distinctive waves: an S wave (positive) illustrating the velocity of apical myocardial movement during ventricular systole, an E’ wave (negative) depicting the velocity of myocardial movement away from the apex during rapid diastolic filling, and an A’ wave (negative) correlating with the velocity of myocardial movement towards basal veins during atrial contraction at the end of diastole. The E wave serves as an indicator of LV myocardial relaxation, calculated as the average of values measured on the septal and lateral sides of the mitral annulus (normal values >8 and >10, respectively).

Combining all these parameters, obtained by PW and TDI doppler, allows us to obtain even more precise estimation of cardiac function, and they could be considered surrogates of AV coupling function estimation. The most used index is the E/e’ ratio, useful for evaluating both the different degrees of diastolic dysfunction and the filling pressures of the left atrium. When the AV junction excursion is affected, the initial decline in the E wave peak results from the diminished elastic capacity of the left ventricle and reduced relaxation forces, concomitant with a reduction in the E’ wave peak (indicative of decreased myocardial movement). However, as left atrium filling pressure rises, a dissociation emerges between the progression of the E wave, which tends to increase, and the consistently decreasing average E’ peak as longitudinal LV function worsens. Consequently, with the gradual compromise of AV coupling, the AV plane excursion diminishes, accompanied by an increase in end-diastolic ventricular and atrial pressures [48]. Ultimately, the modifications of E and E’ waves as well of the E/E’ ratio are expression of the AV junction excursion impairment and of alterated LV filling processes. An E/e’ ratio <8 is considered normal; instead, E/E’ >15 is correlated to severe diastolic dysfunction and increased LA filling pressures: intermediate values suggest the need for integrative parameters.

### 5.2 Strain Imaging

Most recent echocardiographic methods, useful for the AV coupling evaluation, are based on the analysis of the “strain”, i.e., the deformation of an object concerning its starting shape and the speed with which it occurs. The amount of deformation is usually expressed as a percentage. Myocardial ventricular contraction implies, at the same time, myocardial shortening in the longitudinal direction (negative values), torsion and thickening (positive values), all useful parameters for a systolic function evaluation; this results in 3 possible strain analyses, respectively longitudinal, circumferential and radial. Longitudinal ventricular strain is mainly determined by the fibrous plane movement towards the cardiac apex, which could be considered a fixed point. Of the three components of myocardial movement, the longitudinal strain is the one which best describes the systolic ventricular function. The software analyzes the shape of the LV starting from the apical 4-chamber, 2-chamber and 3-chamber views and the extent of their shortening: the more negative the values are, the better the contractility is. The dependence from the angle incidence of the sampling is a strong limitation of TDI. On the contrary, myocardial speckle tracking is angle-independent and allows for the study of all components of regional and global systolic deformation.

**Fig. 2.**
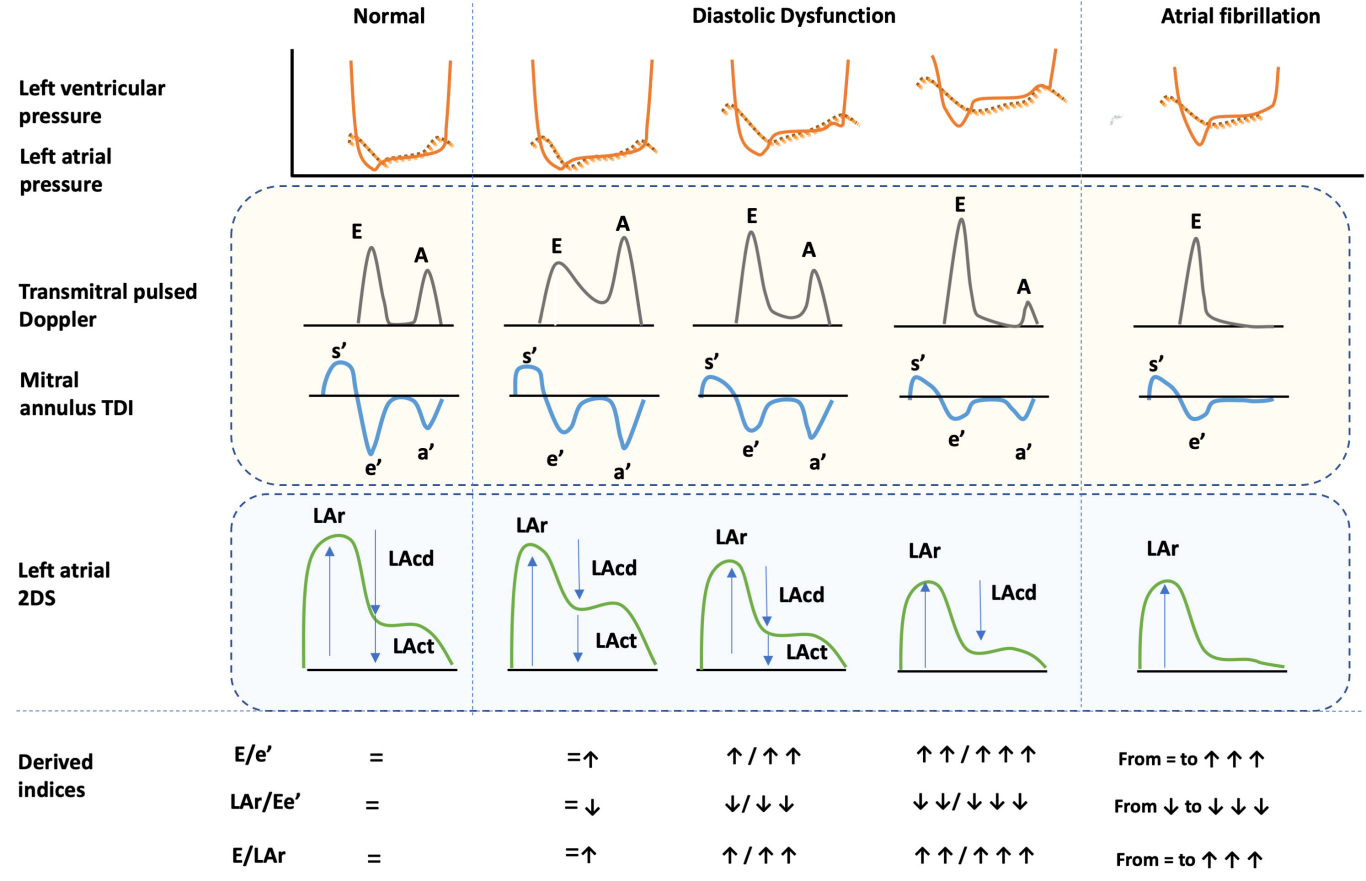
**Doppler and strain measures of diastolic function**. In the figure, Doppler and strain measures of diastolic function are summarized. On the top of the figure, the left atrial (dotted lines) and ventricular (continue line) pressures are represented in normal subject (left), in patients with diastolic dysfunction (middle figure) and in those with atrial fibrillation (right). For each of these groups pulsed and tissue Doppler, and atrial two dimensional strain are also shown. The behavior of the combined measures in the different groups is reported at the bottom of the figure. 2DS, two-dimensional speckle tracking; A, late diastolic wave at pulsed Doppler; a’, late diastolic wave at tissue Doppler; E, early diastolic wave at pulsed Doppler; e’, early diastolic wave at pulsed Doppler; E/e’, ratio between E and e’; E/LAr, ratio between E and LAr; LAcd, left atrial conduit at two-dimensional speckle tracking analysis; LAct, left atrial contraction at two-dimensional speckle tracking analysis; LAr, left atrial reservoir at two-dimensional speckle tracking analysis; LAr/Ee’, ratio between LAr and E/e’; s’, annulus peak systolic velocity; TDI, Tissue Doppler Imaging.

In a completely specular way, for analyzing AV excursion and coupling, the atrial longitudinal strain [49] can also be taken into consideration: in fact, a shortening of the LV during the systole (reservoir phase) corresponds to an enlargement of the LA mediated by the movement of the AV plane towards the cardiac apex; similarly, during diastole, the LV distends and the LA returns to original volume (conduction phase) thanks to the movement of the valve plane from the apex. During the atrial systole (the contraction phase), the AV plane moves away from the cardiac apex, favoring the LV filling and powering the subsequent ventricle contraction (Fig. 2). This movement allows the blood volume to be kept constant during the cardiac cycle, and the specular image of longitudinal strain curves of LA and LV can also demonstrate the synchrony between atrial and ventricular chambers. The atrial longitudinal strain can be obtained in a 4-chamber view, analyzing the region of interest built with the software, considering the AV junction and the atrial bases as points of attention. LA reservoir strain has been associated with elevated filling pressures.

### 5.3 Combined Measures of Pulsed Doppler/TDI and Strain Imaging

**Fig. 3.**
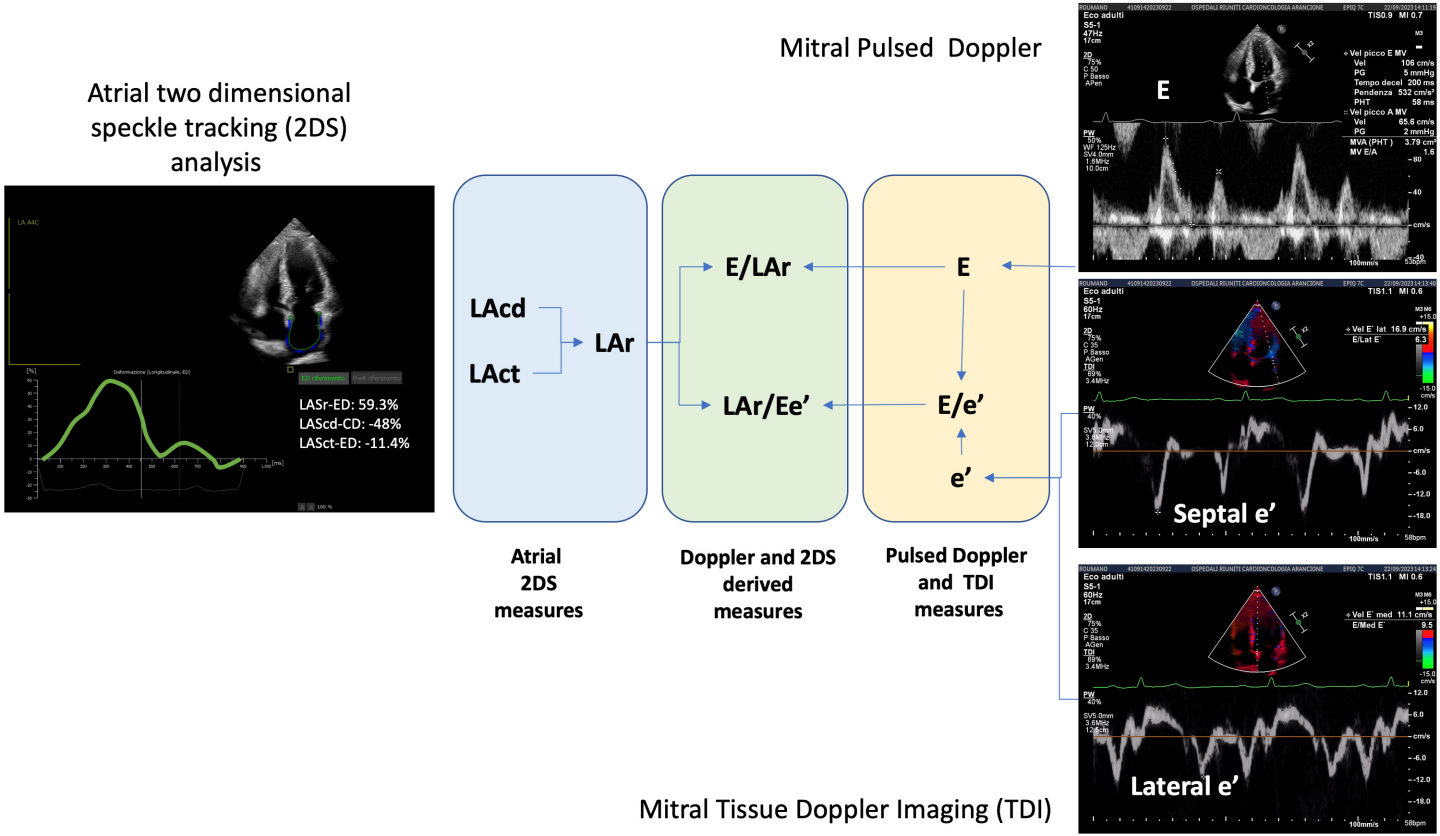
**Echocardiographic measures of diastolic function**. In the figure, echocardiographic assessment of diastolic function by Doppler and strain measures of diastolic function is summarized. Both single and combined measures are represented. 2DS, two dimensional speckle tracking analysis; E, early diastolic wave at pulsed Doppler; e’, early diastolic wave at pulsed Doppler; E/e’, ratio between E and e’; E/LAr, ratio between E and LAr; LAcd (LAScd-CD), left atrial conduit at two-dimensional speckle tracking analysis; LAct (LASct-ED), left atrial contraction at two-dimensional speckle tracking analysis; LAr (LASr-ED), left atrial reservoir at two-dimensional speckle tracking analysis; LAr/Ee’, ratio between LAr andf E/e’; TDI, Tissue Doppler Imaging.

There are new indices that better correlate with filling pressures and could represent surrogates of AV coupling measurement. Branauer *et al*. [50] have investigated the usefulness of an LA filling index, defined as the ratio of the mitral early-diastolic inflow peak velocity (E) over the LA reservoir strain (i.e., E/LA strain ratio). They found that it better correlated with elevated filling pressure and that values >3.27 were significantly associated with the risk of HF hospitalization at two years. Another useful index, recently evaluated in patients affected by stable coronary artery disease and HFpEF, is left atrial strain relaxation (LASr)/Ee’ septal ratio [51]. This parameter agrees with elevated LA filling pressures and invasive LV end-diastolic pressure measurement. Moreover, it better correlates with these parameters than other conventional indices. Both these indices combine the measurement of the AV plane excursion with the assessment of a functional parameter (the LV ventricular inflow), allowing us to better evaluate the AV coupling function (Fig. 3).

## 6. Therapeutic Strategies for Atrial Function and AV Coupling

### 6.1 Atrial Function in Heart Failure

Nowadays, in contrast to HFrEF, which can benefit from several medical therapies to reduce of CV death and hospitalizations, HFpEF lacks effective drugs to improve of CV outcomes. Probably, this could be due to the extremely variegate etiopathogenesis of the disease, which accounts for, among others, arterial hypertension, diabetes, obesity and metabolic syndrome, ischemic disease, kidney failure and fluid retention, pulmonary hypertension and atrial fibrillation. However, all these pathologies promote chronic low-grade inflammation of the heart and the vessels and probably lead to a common pathogenic alteration of cardiac structure and function, characterized by myocardial fibrosis, increased stiffness and elevated filling pressures [52]. HFpEF is currently considered an inflammatory cardio-reno-vascular condition that determines chronic microvascular inflammation and endothelial dysfunction, together with a pronounced deficiency of the nitric oxide (through the cyclic guanosine monophosphate (cGMP)-protein kinase G signaling axis), leading to hypertrophic and/or fibrotic remodeling of the heart and the vessels. As mentioned before, these alterations could lead to an impairment of the normal AV junction excursion, thus ending in AV uncoupling. Nevertheless, novel therapeutical strategies have been studied in the last few years to face this pathogenic cascade.

According to the latest ESC guidelines [53], the mean treatment of HFpEF is the screening and the treatment of cardiovascular and non-cardiovascular comorbidities. Treatment of hypertension, dyslipidemia and diabetes is recommended to prevent HF and hospitalization. Moreover, bad habits (sedentariness, obesity, smoking and alcohol abuse) are discouraged.

Furthermore, the new drug class of sodium-glucose co-transporter-2 inhibitors (SGLT2i) have recently been studied on patients affected by HFpEF. The EMPEROR-Preserved trial (Empaglifozin in Heart Failure with Preserved Ejection Fraction) [54] first (for Empaglifozin) and the DELIVER (Dapaglifozin in Heart Failure with Mildly Reduced or Preserved Ejection Fraction) [55] one then (for Dapaglifozin) have demonstrated SGLT2i to reduce the mortality and HF hospitalizations in a population of patients with HF and LV EF >40%. Besides them, diuretic therapy is recommended to improve symptoms and signs in patients with HFpEF, although a reduction in CV death or hospitalization has never been demonstrated for diuretic therapy.

Recently, a new selective, non-steroidal MRA, the finerenone, has demonstrated the reduction of a composite CV end-point of CV death, non-fatal myocardial infarction, non-fatal stroke or HF hospitalization in patients affected by diabetes and chronic kidney disease (CKD) [56, 57]. By these data, finer one is recommended for HF treatment in patients affected by type 2 diabetes mellitus and CKD.

Moreover, the sub analysis of some previous studies that enrolled patients with LVEF >40% has shown interesting results in HFpEF outcomes. For example, the TOPCAT trial [58] has demonstrated a beneficial effect of spironolactone in a selected pool of patients enrolled in the USA, Canada, Argentina and Brazil, while Candesartan, in the CHARM-preserved trial (Effects of candesartan in patients with chronic heart failure and preserved left-ventricular ejection fraction: the CHARM-Preserved Trial) [59], reduced hospitalization for chronic HF in a patient with HFpEF. In the PARAGON-HF trial [60], Sacubitril-Valsartan (S/V) was beneficial in specific subgroups of patients, i.e., females, EF <57%, estimated glomerular filtration rate (eGFR) <60 mg/mL and high sensitivity Troponin I (TnI-HS) >17 ng/L. Furthermore, recent studies [61] have demonstrated that S/V treatment might improve LV systolic and diastolic function and reverse remodeling in subjects with HFrEF. A significant improvement in LA dimensions and function and changes in LA function (in terms of peak atrial longitudinal strain (PALS)) were proportional to changes in LV EF and RV function after six months of therapy with S/A [62]. Thus, in selected patients, some drugs other than SGLT2i could be used in a comorbidity-guided strategy, and some more could be introduced in the treatment of HFpEF.

A novel field of interest relates to relaxin-2, which could be a new candidate drug for HFpEF. In fact, the anti-fibroproliferative action of relaxin-2 is well described and resides in its ability to counter-act the myofibroblasts transforming growth factor (TGF)-related stimulation, which guides cardiomyocyte development through the secreting of growth factors. Relaxin-2 is also known as a potent vasodilator, inhibiting the stimulation of endothelin-1 gene expression and/or promoting the endothelial pression of its clearance receptor, endothelin type-B receptor. Moreover, it is an inhibitor of chronic vascular inflammation, thus affecting one of the leading mechanisms responsible for HFpEF. It has also been demonstrated that relaxin-2 treatment prevents atrial fibrillation by reversing cardiac fibrosis and increasing sodium current density and conduction velocity in the atria. Some data have shown also a better glucose control in mice treated with relaxin-2. Despite these potential benefits, the recently published RELAX-AHF trial (Effects of Serelaxin in Patients with Acute Heart Failure) has not been demonstrated to reduce CV mortality at 180 days and worsening HF in the first five days in patients affected by acute HF and treated with short-acting recombinant relaxin, Serelaxin. Nevertheless, it demonstrated a short-term HF symptom relief and biomarker improvement. Ongoing studies aim to evaluate the effect of long-acting human relaxin analogue on the cardio-reno-vascular system to possible use in chronic HF [63].

### 6.2 Cardiac Resynchronization Therapy in Prolonged PR Interval

AV coordination can be re-established by restoration of AV coupling. This can lead to improved diastolic filling and higher cardiac output, abolishing premature closing of the mitral valve and increasing the diastolic filling time [64]. The AV coupling is essential, especially in patients with HF, considering the haemodynamic implications in these patient settings. The first studies date back to the era of CRT in the early 1990s. They employed RV pacing, resulting in a significant functional and symptomatic improvement with restored AV coupling in patients with a prolonged PR interval. For example, in the study of Gervais *et al*. [65], RV pacing at an AV delay of 100 ms increased LV EF and blood pressure, decreased the cardiothoracic ratio on chest X-ray, and improved HF symptoms. Brecker *et al*. [66] found a significant reduction in mitral and tricuspid regurgitation duration after AV optimization, with increased LV and RV filling times, and improved cardiac output. Despite the beneficial effect of AV restoring coupling, employing RV pacing can create an intraventricular desynchronization. Thus, some studies were conducted on minimizing RV pacing by prolonging AV conduction times to allow spontaneous ventricular conduction with longer AV conduction times. However, these studies in implantable cardioverter defibrillator (ICD) patients showed higher overall death and HF event rates using this kind of stimulation as compared with ventricular backup pacing.

Despite the increasing attention paid to minimizing RV pacing-induced dyssynchrony, there is a need to avoid induced PR prolongation and inappropriate AV coupling. CRT allows the restoration of inter- and intraventricular synchrony in HF patients with a wide QRS complex [67, 68]. However, CRT restores not only inter- and intraventricular dyssynchrony but also avoids inappropriate AV coupling. In the PATH-CHF study (The Pacing Therapies for Congestive Heart Failure) [69], HF patients with an average PR interval of 210 msec improved EF after restoring AV coupling compared with only biventricular pacing. Two sub-analyses of the COMPANION trial (Cardiac-Resynchronization Therapy with or without an Implantable Defibrillator in Advanced Chronic Heart Failure) showed a reduction in all-cause mortality and HF hospitalization in patients carrying CRT with a prolonged PR interval, compared to ones with a normal PR interval, irrespective of the bundle branch block pattern. Even the sub-analysis of the CARE-HF trial confirmed that shortening the PR interval can improve the prognosis, as can shortening the QRS duration by CRT. Non-left bundle branch block (Non-LBBB) patients were investigated in the MADIT-CRT study. In both sub-analyses, CRT was associated with adverse clinical outcomes in patients with a normal PR interval. These results may be explained because of ventricular desynchronization due to biventricular pacing. However, CRT reduces the risk of HF and all-cause mortality in patients with long PR interval (>230 ms), maybe because the potentially unfavorable effects of biventricular pacing in these patients are overruled by restoration of AV coupling [69, 70].

Other studies showed different results. In patients with normal AV interval, there is a beneficial effect of CRT, rather than in patients with prolonged PR interval (even in patients with LBBB), as shown in a medical registry of patients with an implanted ICD or CRT-D (CRT with ICD devices), thus not demonstrating an association between prolonged PR interval and a reduction in HF hospitalization or death [71]. Similar results were shown in a Mayo Clinic study in patients with CRT implantation. Indeed, they found that the CRT response rate was better in patients with normal PR intervals than in those with prolonged PR intervals.

In conclusion, patients with prolonged PR interval seem to have a worse prognosis than patients with normal PR interval. Therefore, this setting of patients may benefit from normalizing AV conduction times by CRT, thus improving AV coupling.

### 6.3 Surgical Aspects

The model of a constant-volume heart pump and the consequent AV coupling concept could significantly impact surgical procedures and the projecting of mitral prosthesis. It is known that the subvalvular apparatus contributes to LV performance significantly, not only by maintaining the physiologic valve continence function [72]. Several cases of ventricular systolic dysfunction that occurred after cutting the second-order chordae tendineae are well described in the literature. This effect has been attributed to the role of the second-order chordae tendineae in preserving the normal LV shape and in unloading LV wall tension forces. We think, however, that the subvalvular apparatus plays an essential role in stabilizing the mitral valve during the AV plane motion towards the apex in the systole phase. Therefore, by creating some fixed traction points, the whole AV circumference and the leaflets can be moved towards the apex thanks to the contraction of the LV and of the papillary muscles. These considerations suggest cutting the chordae tendineae should be avoided whenever possible during surgical mitral valve repair or replacement. Moreover, the design of mitral valve prosthesis should consider these aspects to spare the subvalvular apparatus and preserve its functional role in ventricular systole.

## 7. Conclusions

Left atrial and ventricular function are strictly linked to each other, and both contribute to global heart performance, according to the constant-volume pump function model. The interplay between the two chambers is defined as AV coupling. Conditions that compromise the atrial and the ventricular function, as well as the AV coupling, may alter the heart function and are associated with a worse prognosis and heart failure. Novel echocardiographic methods, i.e., atrial and ventricular strain measurement, allow a more accurate evaluation and an early diagnosis of AV dysfunction and uncoupling. These abnormalities are found in many cases of HFpEF, a variegate group of cardiac affections characterized by HF symptoms but LVEF >50%. There is growing attention on new specific therapies for HFpEF, and the early identification of the hemodynamic or electrical uncoupling phenotype allows us to define a more tailored therapeutic strategy. However, the precise relationship between AV coupling impairment and heart pathologies remains uncertain. Distinguishing between systolic and diastolic functions in various heart failure scenarios can be challenging. Further research is necessary to accurately delineate the respective contributions of atrial and ventricular function, as well as AV coupling, to the spectrum of heart conditions.

## Abbreviations

AV, atrioventricular; CKD, chronic kidney disease; CV, cardiovascular; CRT, cardiac resynchronization therapy; EF, ejection fraction; GLS, global longitudinal strain; HF, heart failure; HFpEF, heart failure with preserved ejection fraction; HFrEF, heart failure with reduced ejection fraction; ICD, implantable cardioverter defibrillator; LA, left atrium; LV, left ventricle; MAPSE, mitral annular plane systolic excursion; RV, right ventricle; TDI, Tissue Doppler Imaging.
